# *N*,*N*′-(Methyl­enedi-*p*-phenyl­ene)dibenzamide

**DOI:** 10.1107/S1600536809031304

**Published:** 2009-08-12

**Authors:** Sohail Saeed, Naghmana Rashid, Rizwan Hussain, Peter G. Jones

**Affiliations:** a Department of Chemistry, Research Complex, Allama Iqbal Open University, Islamabad, Pakistan; b Directorate of Chemical & Power Sources, National Development Complex, PO Box 2216, Islamabad, Pakistan; c Institut für Anorganische und Analytische Chemie, Technische Universität Braunschweig, Postfach 3329, 38023 Braunschweig, Germany

## Abstract

The title compound, C_27_H_22_N_2_O_2_, consists of two chemically equivalent halves. However, it displays no crystallographic symmetry, only an approximate local twofold symmetry (r.m.s. deviation = 0.15 Å between the two halves of the molecule) is observed. In the crystal, mol­ecules are connected by two anti­parallel classical N—H⋯O hydrogen bonds, forming broad chains parallel to (10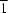). A series of weak C—H⋯N/O hydrogen bonds is also present.

## Related literature

For general background to the chemistry of polymers and polyamides, see Ataei *et al.* (2005 ▶); Yang *et al.* (2002 ▶). For related structures, see: Im & Jung (2000 ▶).
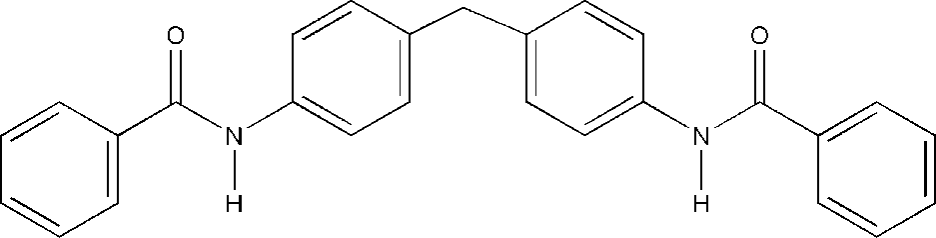


## Experimental

### 

#### Crystal data

C_27_H_22_N_2_O_2_
*M* *_r_* = 406.47
Triclinic, 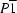

*a* = 5.7296 (7) Å
*b* = 9.601 (1) Å
*c* = 20.045 (2) Å
α = 88.517 (8)°
β = 82.293 (8)°
γ = 75.67 (1)°
*V* = 1058.7 (2) Å^3^
*Z* = 2
Cu *K*α radiation
μ = 0.64 mm^−1^
*T* = 100 K
0.20 × 0.10 × 0.04 mm

#### Data collection

Oxford Diffraction Xcalibur Nova A diffractometer
Absorption correction: multi-scan (CrysAlis Pro; Oxford Diffraction, 2009 ▶) *T*_min_ = 0.781, *T*_max_ = 1.000 (expected range = 0.762–0.975)
14884 measured reflections
4355 independent reflections
3819 reflections with *I* > 2σ(*I*)
*R*_int_ = 0.026

#### Refinement

*R*[*F* ^2^ > 2σ(*F* ^2^)] = 0.038
*wR*(*F* ^2^) = 0.102
*S* = 1.07
4355 reflections
288 parameters
H atoms treated by a mixture of independent and constrained refinement
Δρ_max_ = 0.16 e Å^−3^
Δρ_min_ = −0.18 e Å^−3^

### 

Data collection: *CrysAlis Pro* (Oxford Diffraction, 2009 ▶); cell refinement: *CrysAlis Pro*; data reduction: *CrysAlis Pro*; program(s) used to solve structure: *SHELXS97* (Sheldrick, 2008 ▶); program(s) used to refine structure: *SHELXL97* (Sheldrick, 2008 ▶); molecular graphics: *XP* (Siemens, 1994 ▶); software used to prepare material for publication: *SHELXL97*.

## Supplementary Material

Crystal structure: contains datablocks I, global. DOI: 10.1107/S1600536809031304/im2132sup1.cif

Structure factors: contains datablocks I. DOI: 10.1107/S1600536809031304/im2132Isup2.hkl

Additional supplementary materials:  crystallographic information; 3D view; checkCIF report

## Figures and Tables

**Table 1:** Hydrogen-bond geometry (Å, °)

*D*—H⋯*A*	*D*—H	H⋯*A*	*D*⋯*A*	*D*—H⋯*A*
N1—H01⋯O2^i^	0.875 (17)	2.017 (17)	2.8745 (13)	166.3 (14)
N2—H02⋯O1^ii^	0.876 (16)	2.088 (16)	2.9358 (12)	162.7 (14)
C16—H16⋯O2^i^	0.95	2.60	3.3090 (14)	132
C35—H35⋯N1^i^	0.95	2.72	3.5459 (15)	146
C33—H33⋯O1^ii^	0.95	2.43	3.1913 (15)	137
C42—H42⋯O1^ii^	0.95	2.58	3.3003 (14)	133

Symmetry codes: (i) 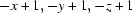; (ii) 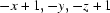.

**Fig. 1.**
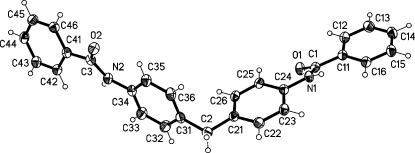
Thermal ellipsoid plot (50% probability level) of the title compound.

**Fig. 2.**
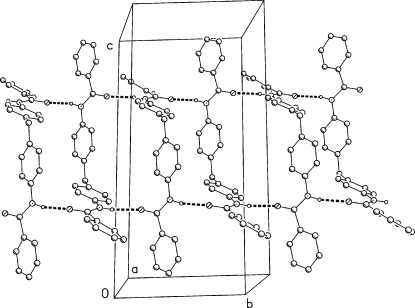
Packing diagram viewed parpendicular to (101). Classical H bonds are indicated by thick dashed lines. H atoms not involved in these H bonds are omitted for clarity.

## supplementary crystallographic information

### Comment

High-temperature polymers have received much attention because of increasing demands for the replacement of ceramics and metals (Ataei *et al.*, 2005). However, in many cases they are insoluble and do not melt below their decomposition temperature, which restricts their applications (Im & Jung, 2000). Many studies have therefore focused on obtaining aromatic polymers that are processable by conventional techniques (Yang *et al.*, 2002). The title compound is a logical precursor for an attempt to synthesize polyamides and polyimides having excellent thermal and mechanical properties.

The molecule of the title compound is shown in Fig. 1. Molecular dimensions may be regarded as normal, as may the *trans* geometry at the amide groups. The molecule possesses no crystallographic symmetry, but displays approximate twofold symmetry with a r.m.s. deviation of 0.15 Å between the two halves of the molecule. There are however significant differences between torsion angles of chemically equivalent amide groups, *e.g.* C1—N1—C24—C25 - 31.5 (2) *versus*. C3—N2—C34—C35 - 16.2 (2)°. The outer pairs of rings are approximately parallel [interplanar angles: C11–16/C21–26 2.91 (7), C31–36/C41–46 10.72 (6)°] whereas the central pair of rings are approximately perpendicular [C21–26/C31–36 84.02 (4)°].

The main features of the molecular packing are the classical H bonds of the N—H···OC type, which are mutually antiparallel and link the molecules to form broad chains parallel to (101) (Fig. 2, Table 1). A series of narrow-angled (C—H···N/O 132–146°) weak H bonds are probably of less structural significance (Table 1).

### Experimental

All reagents and organic solvents were of analytical grade and commercially available. The title compound was accidentally generated during the reaction of 4,4'- diaminodiphenylmethane with 2-thiophene-carbonyl chloride; it was isolated from the reaction mixture by column chromatography in 30% yield and then purified by recrystallization from ethanol. Colourless single crystals suitable for X-ray analysis were obtained after one week by slow evaporation from an ethanolic solution. Crystals formed as thin plates or somewhat thicker laths; both proved to have the same cell constants.

### Refinement

NH H atoms were refined freely. Other H atoms were placed in calculated positions and refined using a riding model with C—H_arom_ 0.95 Å, *C*—H_methylene_ 0.99 Å; these hydrogen *U* values were fixed at 1.2 × *U*(eq) of the parent atom. Data are 99.6% complete to 2θ 145°.

### Crystal data

**Table:**

C_27_H_22_N_2_O_2_	*F*(000) = 428
*M**_r_* = 406.47	*D*_x_ = 1.275 Mg m^−^^3^
Triclinic, *P*1	Melting point: 449 K
*a* = 5.7296 (7) Å	Cu *K*α radiation, λ = 1.54184 Å
*b* = 9.601 (1) Å	Cell parameters from 10160 reflections
*c* = 20.045 (2) Å	θ = 3.3–75.8°
α = 88.517 (8)°	µ = 0.64 mm^−^^1^
β = 82.293 (8)°	*T* = 100 K
γ = 75.67 (1)°	Lath, colourless
*V* = 1058.7 (2) Å^3^	0.20 × 0.10 × 0.04 mm
*Z* = 2	

### Data collection

**Table:**

Oxford Diffraction Xcalibur Nova A diffractometer	4355 independent reflections
Radiation source: Nova (Cu) X-ray Source	3819 reflections with *I* > 2σ(*I*)
mirror	*R*_int_ = 0.026
Detector resolution: 10.3543 pixels mm^-1^	θ_max_ = 76.0°, θ_min_ = 4.5°
ω scans	*h* = −7→7
Absorption correction: multi-scan (*CrysAlis PRO*; Oxford Diffraction, 2009)	*k* = −12→10
*T*_min_ = 0.781, *T*_max_ = 1.000	*l* = −25→24
14884 measured reflections	

### Refinement

**Table:**

Refinement on *F*^2^	Primary atom site location: structure-invariant direct methods
Least-squares matrix: full	Secondary atom site location: difference Fourier map
*R*[*F*^2^ > 2σ(*F*^2^)] = 0.038	Hydrogen site location: inferred from neighbouring sites
*wR*(*F*^2^) = 0.102	H atoms treated by a mixture of independent and constrained refinement
*S* = 1.07	*w* = 1/[σ^2^(*F*_o_^2^) + (0.0552*P*)^2^ + 0.2168*P*] where *P* = (*F*_o_^2^ + 2*F*_c_^2^)/3
4355 reflections	(Δ/σ)_max_ = 0.001
288 parameters	Δρ_max_ = 0.16 e Å^−^^3^
0 restraints	Δρ_min_ = −0.18 e Å^−^^3^

### Special details

**Table:**

Geometry. All e.s.d.'s (except the e.s.d. in the dihedral angle between two l.s. planes) are estimated using the full covariance matrix. The cell e.s.d.'s are taken into account individually in the estimation of e.s.d.'s in distances, angles and torsion angles; correlations between e.s.d.'s in cell parameters are only used when they are defined by crystal symmetry. An approximate (isotropic) treatment of cell e.s.d.'s is used for estimating e.s.d.'s involving l.s. planes.
Refinement. Refinement of *F*^2^ against ALL reflections. The weighted *R*-factor *wR* and goodness of fit *S* are based on *F*^2^, conventional *R*-factors *R* are based on *F*, with *F* set to zero for negative *F*^2^. The threshold expression of *F*^2^ > σ(*F*^2^) is used only for calculating *R*-factors(gt) *etc*. and is not relevant to the choice of reflections for refinement. *R*-factors based on *F*^2^ are statistically about twice as large as those based on *F*, and *R*- factors based on ALL data will be even larger.Least-squares planes (*x*,*y*,*z* in crystal coordinates) and deviations from them (* indicates atom used to define plane)- 2.5047 (0.0026) *x* + 7.1432 (0.0034) *y* + 2.4159 (0.0097) *z* = 0.1119 (0.0034)* -0.0003 (0.0008) C11 * -0.0057 (0.0008) C12 * 0.0066 (0.0009) C13 * -0.0015 (0.0009) C14 * -0.0045 (0.0008) C15 * 0.0054 (0.0008) C16 - 0.0844 (0.0017) C1 - 0.5968 (0.0020) O1 0.4205 (0.0020) N1Rms deviation of fitted atoms = 0.0046- 2.3282 (0.0026) *x* + 7.4133 (0.0033) *y* + 1.8134 (0.0088) *z* = 0.5814 (0.0051)Angle to previous plane (with approximate e.s.d.) = 2.91 (0.07)* -0.0079 (0.0008) C21 * 0.0076 (0.0008) C22 * 0.0009 (0.0008) C23 * -0.0090 (0.0008) C24 * 0.0085 (0.0008) C25 * -0.0001 (0.0008) C26 0.0290 (0.0016) N1 - 0.0695 (0.0019) C2Rms deviation of fitted atoms = 0.00673.4319 (0.0022) *x* + 3.8138 (0.0049) *y* + 16.6363 (0.0061) *z* = 13.6255 (0.0031)Angle to previous plane (with approximate e.s.d.) = 84.02 (0.04)* 0.0014 (0.0008) C31 * -0.0008 (0.0009) C32 * -0.0014 (0.0009) C33 * 0.0030 (0.0008) C34 * -0.0024 (0.0008) C35 * 0.0002 (0.0008) C36 - 0.0109 (0.0016) N2 0.0150 (0.0018) C2Rms deviation of fitted atoms = 0.00182.8287 (0.0024) *x* + 4.9963 (0.0040) *y* + 16.4884 (0.0060) *z* = 14.1826 (0.0051)Angle to previous plane (with approximate e.s.d.) = 10.72 (0.06)* -0.0120 (0.0008) C41 * 0.0057 (0.0008) C42 * 0.0049 (0.0008) C43 * -0.0092 (0.0008) C44 * 0.0029 (0.0008) C45 * 0.0078 (0.0008) C46 - 0.0326 (0.0017) C3 0.3984 (0.0019) O2 - 0.5360 (0.0020) N2Rms deviation of fitted atoms = 0.0077

### Fractional atomic coordinates and isotropic or equivalent isotropic displacement parameters (Å^2^)

**Table:**

	*x*	*y*	*z*	*U*_iso_*/*U*_eq_	
O1	0.76324 (16)	0.11312 (8)	0.25609 (4)	0.0293 (2)	
O2	−0.05166 (15)	0.43352 (8)	0.76182 (4)	0.02817 (19)	
N1	0.91641 (18)	0.30294 (10)	0.27475 (5)	0.0241 (2)	
H01	0.958 (3)	0.3787 (17)	0.2567 (8)	0.034 (4)*	
N2	0.15925 (18)	0.20072 (10)	0.73950 (5)	0.0237 (2)	
H02	0.156 (3)	0.1111 (18)	0.7476 (8)	0.033 (4)*	
C1	0.8376 (2)	0.21803 (11)	0.23510 (6)	0.0234 (2)	
C2	0.9989 (2)	0.24643 (14)	0.55736 (6)	0.0294 (3)	
H2A	1.1296	0.1591	0.5625	0.035*	
H2B	1.0546	0.3298	0.5712	0.035*	
C3	−0.0255 (2)	0.30332 (11)	0.77064 (5)	0.0231 (2)	
C11	0.8434 (2)	0.25660 (11)	0.16191 (6)	0.0239 (2)	
C12	0.6816 (2)	0.21104 (13)	0.12666 (6)	0.0291 (3)	
H12	0.5695	0.1623	0.1497	0.035*	
C13	0.6834 (2)	0.23651 (14)	0.05825 (6)	0.0340 (3)	
H13	0.5705	0.2068	0.0347	0.041*	
C14	0.8497 (2)	0.30526 (13)	0.02406 (6)	0.0330 (3)	
H14	0.8522	0.3216	−0.0229	0.040*	
C15	1.0122 (2)	0.35010 (12)	0.05876 (6)	0.0297 (3)	
H15	1.1265	0.3968	0.0354	0.036*	
C16	1.0087 (2)	0.32696 (12)	0.12763 (6)	0.0255 (2)	
H16	1.1189	0.3591	0.1513	0.031*	
C21	0.9656 (2)	0.26234 (12)	0.48346 (6)	0.0245 (2)	
C22	1.1219 (2)	0.32417 (13)	0.44001 (6)	0.0275 (2)	
H22	1.2421	0.3597	0.4577	0.033*	
C23	1.1061 (2)	0.33505 (12)	0.37156 (6)	0.0270 (2)	
H23	1.2157	0.3768	0.3428	0.032*	
C24	0.9296 (2)	0.28478 (11)	0.34494 (5)	0.0228 (2)	
C25	0.7677 (2)	0.22582 (12)	0.38770 (6)	0.0249 (2)	
H25	0.6438	0.1936	0.3703	0.030*	
C26	0.7884 (2)	0.21443 (12)	0.45618 (6)	0.0260 (2)	
H26	0.6787	0.1729	0.4850	0.031*	
C31	0.7762 (2)	0.23612 (13)	0.60485 (5)	0.0257 (2)	
C32	0.7603 (2)	0.10857 (14)	0.63724 (7)	0.0333 (3)	
H32	0.8919	0.0259	0.6290	0.040*	
C33	0.5569 (2)	0.09899 (13)	0.68137 (6)	0.0314 (3)	
H33	0.5504	0.0104	0.7028	0.038*	
C34	0.3625 (2)	0.21848 (12)	0.69433 (5)	0.0227 (2)	
C35	0.3748 (2)	0.34678 (12)	0.66207 (6)	0.0279 (2)	
H35	0.2428	0.4293	0.6701	0.033*	
C36	0.5802 (2)	0.35433 (12)	0.61812 (6)	0.0282 (3)	
H36	0.5867	0.4428	0.5966	0.034*	
C41	−0.2063 (2)	0.24956 (11)	0.81921 (5)	0.0230 (2)	
C42	−0.1489 (2)	0.11654 (12)	0.85073 (6)	0.0258 (2)	
H42	0.0112	0.0567	0.8422	0.031*	
C43	−0.3239 (2)	0.07097 (12)	0.89452 (6)	0.0285 (3)	
H43	−0.2834	−0.0199	0.9157	0.034*	
C44	−0.5577 (2)	0.15796 (13)	0.90741 (6)	0.0289 (3)	
H44	−0.6783	0.1261	0.9367	0.035*	
C45	−0.6148 (2)	0.29195 (13)	0.87733 (6)	0.0292 (3)	
H45	−0.7741	0.3524	0.8867	0.035*	
C46	−0.4400 (2)	0.33797 (12)	0.83371 (6)	0.0266 (2)	
H46	−0.4799	0.4301	0.8136	0.032*	

### Atomic displacement parameters (Å^2^)

**Table:**

	*U*^11^	*U*^22^	*U*^33^	*U*^12^	*U*^13^	*U*^23^
O1	0.0409 (5)	0.0230 (4)	0.0258 (4)	−0.0147 (3)	0.0015 (3)	0.0001 (3)
O2	0.0323 (4)	0.0205 (4)	0.0313 (4)	−0.0085 (3)	0.0007 (3)	0.0014 (3)
N1	0.0306 (5)	0.0204 (4)	0.0223 (5)	−0.0097 (4)	−0.0008 (4)	0.0027 (3)
N2	0.0295 (5)	0.0189 (4)	0.0236 (5)	−0.0094 (4)	−0.0005 (4)	0.0019 (3)
C1	0.0245 (5)	0.0195 (5)	0.0247 (5)	−0.0049 (4)	0.0012 (4)	−0.0004 (4)
C2	0.0259 (6)	0.0382 (6)	0.0256 (6)	−0.0111 (5)	−0.0035 (4)	0.0054 (5)
C3	0.0277 (6)	0.0213 (5)	0.0219 (5)	−0.0082 (4)	−0.0046 (4)	0.0006 (4)
C11	0.0269 (5)	0.0187 (5)	0.0242 (5)	−0.0038 (4)	−0.0001 (4)	−0.0004 (4)
C12	0.0298 (6)	0.0295 (6)	0.0285 (6)	−0.0095 (5)	−0.0011 (5)	−0.0003 (4)
C13	0.0358 (7)	0.0386 (7)	0.0290 (6)	−0.0099 (5)	−0.0076 (5)	−0.0012 (5)
C14	0.0412 (7)	0.0324 (6)	0.0230 (6)	−0.0058 (5)	−0.0026 (5)	0.0014 (5)
C15	0.0350 (6)	0.0254 (5)	0.0267 (6)	−0.0071 (5)	0.0024 (5)	0.0027 (4)
C16	0.0278 (6)	0.0219 (5)	0.0261 (6)	−0.0058 (4)	−0.0016 (4)	0.0003 (4)
C21	0.0235 (5)	0.0238 (5)	0.0245 (5)	−0.0038 (4)	−0.0010 (4)	0.0020 (4)
C22	0.0258 (6)	0.0308 (6)	0.0283 (6)	−0.0114 (5)	−0.0043 (4)	0.0042 (4)
C23	0.0275 (6)	0.0281 (6)	0.0266 (6)	−0.0114 (5)	−0.0001 (4)	0.0052 (4)
C24	0.0258 (5)	0.0177 (5)	0.0231 (5)	−0.0032 (4)	−0.0011 (4)	0.0013 (4)
C25	0.0242 (5)	0.0258 (5)	0.0251 (5)	−0.0081 (4)	−0.0005 (4)	−0.0007 (4)
C26	0.0251 (5)	0.0274 (5)	0.0251 (5)	−0.0091 (4)	0.0028 (4)	0.0017 (4)
C31	0.0283 (6)	0.0318 (6)	0.0196 (5)	−0.0119 (5)	−0.0039 (4)	0.0028 (4)
C32	0.0309 (6)	0.0291 (6)	0.0348 (6)	−0.0025 (5)	0.0030 (5)	0.0057 (5)
C33	0.0356 (7)	0.0234 (5)	0.0332 (6)	−0.0075 (5)	0.0016 (5)	0.0071 (4)
C34	0.0267 (5)	0.0235 (5)	0.0197 (5)	−0.0100 (4)	−0.0019 (4)	−0.0006 (4)
C35	0.0335 (6)	0.0230 (5)	0.0259 (6)	−0.0074 (4)	0.0010 (5)	0.0006 (4)
C36	0.0376 (6)	0.0238 (5)	0.0246 (5)	−0.0125 (5)	−0.0009 (5)	0.0027 (4)
C41	0.0273 (6)	0.0222 (5)	0.0211 (5)	−0.0090 (4)	−0.0032 (4)	−0.0015 (4)
C42	0.0286 (6)	0.0231 (5)	0.0248 (5)	−0.0065 (4)	−0.0005 (4)	−0.0005 (4)
C43	0.0354 (6)	0.0236 (5)	0.0264 (6)	−0.0095 (5)	0.0000 (5)	0.0022 (4)
C44	0.0305 (6)	0.0332 (6)	0.0251 (6)	−0.0142 (5)	0.0018 (5)	−0.0017 (5)
C45	0.0254 (6)	0.0319 (6)	0.0293 (6)	−0.0060 (5)	−0.0016 (5)	−0.0020 (5)
C46	0.0299 (6)	0.0244 (5)	0.0253 (5)	−0.0065 (4)	−0.0037 (4)	0.0009 (4)

### Geometric parameters (Å, °)

**Table:**

O1—C1	1.2310 (14)	C41—C46	1.3941 (16)
O2—C3	1.2325 (14)	C41—C42	1.3945 (16)
N1—C1	1.3462 (15)	C42—C43	1.3888 (16)
N1—C24	1.4230 (14)	C43—C44	1.3868 (17)
N2—C3	1.3485 (15)	C44—C45	1.3891 (17)
N2—C34	1.4188 (14)	C45—C46	1.3880 (17)
C1—C11	1.5018 (15)	N1—H01	0.875 (17)
C2—C31	1.5086 (16)	N2—H02	0.876 (16)
C2—C21	1.5180 (15)	C2—H2A	0.9900
C3—C41	1.5021 (15)	C2—H2B	0.9900
C11—C12	1.3937 (17)	C12—H12	0.9500
C11—C16	1.3943 (16)	C13—H13	0.9500
C12—C13	1.3858 (17)	C14—H14	0.9500
C13—C14	1.3878 (19)	C15—H15	0.9500
C14—C15	1.3872 (18)	C16—H16	0.9500
C15—C16	1.3908 (16)	C22—H22	0.9500
C21—C26	1.3907 (16)	C23—H23	0.9500
C21—C22	1.3933 (16)	C25—H25	0.9500
C22—C23	1.3866 (16)	C26—H26	0.9500
C23—C24	1.3932 (16)	C32—H32	0.9500
C24—C25	1.3918 (15)	C33—H33	0.9500
C25—C26	1.3928 (16)	C35—H35	0.9500
C31—C36	1.3874 (17)	C36—H36	0.9500
C31—C32	1.3884 (17)	C42—H42	0.9500
C32—C33	1.3861 (17)	C43—H43	0.9500
C33—C34	1.3889 (17)	C44—H44	0.9500
C34—C35	1.3892 (16)	C45—H45	0.9500
C35—C36	1.3882 (17)	C46—H46	0.9500
			
C1—N1—C24	125.97 (9)	C1—N1—H01	118.0 (10)
C3—N2—C34	128.27 (9)	C24—N1—H01	116.0 (10)
O1—C1—N1	123.28 (11)	C3—N2—H02	117.2 (10)
O1—C1—C11	119.98 (10)	C34—N2—H02	114.5 (10)
N1—C1—C11	116.74 (9)	C31—C2—H2A	108.4
C31—C2—C21	115.57 (10)	C21—C2—H2A	108.4
O2—C3—N2	124.60 (10)	C31—C2—H2B	108.4
O2—C3—C41	119.96 (10)	C21—C2—H2B	108.4
N2—C3—C41	115.44 (9)	H2A—C2—H2B	107.4
C12—C11—C16	119.42 (11)	C13—C12—H12	119.8
C12—C11—C1	116.81 (10)	C11—C12—H12	119.8
C16—C11—C1	123.66 (10)	C12—C13—H13	119.9
C13—C12—C11	120.35 (11)	C14—C13—H13	119.9
C12—C13—C14	120.16 (12)	C15—C14—H14	120.1
C15—C14—C13	119.78 (11)	C13—C14—H14	120.1
C14—C15—C16	120.33 (11)	C14—C15—H15	119.8
C15—C16—C11	119.94 (11)	C16—C15—H15	119.8
C26—C21—C22	117.76 (10)	C15—C16—H16	120.0
C26—C21—C2	122.95 (10)	C11—C16—H16	120.0
C22—C21—C2	119.26 (10)	C23—C22—H22	119.3
C23—C22—C21	121.45 (11)	C21—C22—H22	119.3
C22—C23—C24	120.02 (10)	C22—C23—H23	120.0
C25—C24—C23	119.47 (10)	C24—C23—H23	120.0
C25—C24—N1	122.82 (10)	C24—C25—H25	120.2
C23—C24—N1	117.64 (10)	C26—C25—H25	120.2
C24—C25—C26	119.59 (10)	C21—C26—H26	119.2
C21—C26—C25	121.68 (10)	C25—C26—H26	119.2
C36—C31—C32	117.52 (11)	C33—C32—H32	119.2
C36—C31—C2	121.11 (11)	C31—C32—H32	119.2
C32—C31—C2	121.37 (11)	C32—C33—H33	119.9
C33—C32—C31	121.54 (11)	C34—C33—H33	119.9
C32—C33—C34	120.24 (11)	C36—C35—H35	120.0
C33—C34—C35	118.99 (11)	C34—C35—H35	120.0
C33—C34—N2	117.27 (10)	C31—C36—H36	119.1
C35—C34—N2	123.72 (10)	C35—C36—H36	119.1
C36—C35—C34	119.93 (11)	C43—C42—H42	119.8
C31—C36—C35	121.77 (11)	C41—C42—H42	119.8
C46—C41—C42	119.09 (10)	C44—C43—H43	119.9
C46—C41—C3	118.15 (10)	C42—C43—H43	119.9
C42—C41—C3	122.75 (10)	C43—C44—H44	120.1
C43—C42—C41	120.47 (11)	C45—C44—H44	120.1
C44—C43—C42	120.11 (11)	C46—C45—H45	119.9
C43—C44—C45	119.71 (11)	C44—C45—H45	119.9
C46—C45—C44	120.30 (11)	C45—C46—H46	119.9
C45—C46—C41	120.28 (11)	C41—C46—H46	119.9
			
C24—N1—C1—O1	0.93 (18)	C2—C21—C26—C25	−177.65 (10)
C24—N1—C1—C11	−179.06 (10)	C24—C25—C26—C21	0.86 (17)
C34—N2—C3—O2	3.43 (18)	C21—C2—C31—C36	−70.56 (14)
C34—N2—C3—C41	−176.26 (10)	C21—C2—C31—C32	109.90 (13)
O1—C1—C11—C12	24.81 (15)	C36—C31—C32—C33	−0.13 (19)
N1—C1—C11—C12	−155.20 (11)	C2—C31—C32—C33	179.42 (12)
O1—C1—C11—C16	−151.47 (11)	C31—C32—C33—C34	−0.1 (2)
N1—C1—C11—C16	28.53 (15)	C32—C33—C34—C35	0.48 (19)
C16—C11—C12—C13	−0.57 (17)	C32—C33—C34—N2	179.41 (11)
C1—C11—C12—C13	−177.01 (10)	C3—N2—C34—C33	164.96 (11)
C11—C12—C13—C14	1.23 (19)	C3—N2—C34—C35	−16.17 (18)
C12—C13—C14—C15	−0.82 (19)	C33—C34—C35—C36	−0.58 (17)
C13—C14—C15—C16	−0.24 (18)	N2—C34—C35—C36	−179.43 (10)
C14—C15—C16—C11	0.89 (17)	C32—C31—C36—C35	0.03 (18)
C12—C11—C16—C15	−0.49 (16)	C2—C31—C36—C35	−179.52 (11)
C1—C11—C16—C15	175.69 (10)	C34—C35—C36—C31	0.32 (18)
C31—C2—C21—C26	−25.19 (16)	O2—C3—C41—C46	23.81 (15)
C31—C2—C21—C22	156.51 (11)	N2—C3—C41—C46	−156.49 (10)
C26—C21—C22—C23	−1.41 (17)	O2—C3—C41—C42	−155.33 (11)
C2—C21—C22—C23	176.98 (11)	N2—C3—C41—C42	24.37 (15)
C21—C22—C23—C24	0.60 (18)	C46—C41—C42—C43	1.81 (16)
C22—C23—C24—C25	0.97 (17)	C3—C41—C42—C43	−179.06 (10)
C22—C23—C24—N1	177.98 (10)	C41—C42—C43—C44	−0.21 (17)
C1—N1—C24—C25	−31.50 (17)	C42—C43—C44—C45	−1.20 (18)
C1—N1—C24—C23	151.60 (11)	C43—C44—C45—C46	1.01 (18)
C23—C24—C25—C26	−1.68 (16)	C44—C45—C46—C41	0.60 (17)
N1—C24—C25—C26	−178.53 (10)	C42—C41—C46—C45	−2.00 (16)
C22—C21—C26—C25	0.67 (17)	C3—C41—C46—C45	178.83 (10)

### Hydrogen-bond geometry (Å, °)

**Table:**

*D*—H···*A*	*D*—H	H···*A*	*D*···*A*	*D*—H···*A*
N1—H01···O2^i^	0.875 (17)	2.017 (17)	2.8745 (13)	166.3 (14)
N2—H02···O1^ii^	0.876 (16)	2.088 (16)	2.9358 (12)	162.7 (14)
C16—H16···O2^i^	0.95	2.60	3.3090 (14)	132
C35—H35···N1^i^	0.95	2.72	3.5459 (15)	146
C33—H33···O1^ii^	0.95	2.43	3.1913 (15)	137
C42—H42···O1^ii^	0.95	2.58	3.3003 (14)	133

Symmetry codes: (i) −*x*+1, −*y*+1, −*z*+1; (ii) −*x*+1, −*y*, −*z*+1.

## References

[bb1] Ataei, S. M., Sarrafi, Y., Hatami, M. & Faizi, L. A. (2005). *Eur. Polym. J.* **41**, 491–499.

[bb2] Im, J. K. & Jung, J. C. (2000). *Polymers*, **41**, 8709–8716.

[bb3] Oxford Diffraction (2009). *CrysAlis Pro* Oxford Diffraction Ltd, Yarnton, Oxfordshire, England.

[bb4] Sheldrick, G. M. (2008). *Acta Cryst.* A**64**, 112–122. 10.1107/S0108767307043930 18156677

[bb5] Siemens (1994). *XP* Siemens Analytical X-ray Instruments Inc., Madison, Wisconsin, USA.

[bb6] Yang, C.-P., Chen, R.-S. & Hsu, M.-F. (2002). *J. Polym. Res.* **9**, 245–250.

